# Targeted Delivery of Lovastatin and Tocotrienol to Fracture Site Promotes Fracture Healing in Osteoporosis Model: Micro-Computed Tomography and Biomechanical Evaluation

**DOI:** 10.1371/journal.pone.0115595

**Published:** 2014-12-19

**Authors:** Nurul ‘Izzah Ibrahim, Mohd Fadhli Khamis, Mohd Faridz Mod Yunoh, Shahrum Abdullah, Norazlina Mohamed, Ahmad Nazrun Shuid

**Affiliations:** 1 Department of Pharmacology, Faculty of Medicine, Universiti Kebangsaan Malaysia Medical Centre, Jalan Yaacob Latif, Bandar Tun Razak, Cheras, Kuala Lumpur, Malaysia; 2 School of Dental Sciences, Universiti Sains Malaysia, Kubang Kerian, Kelantan, Malaysia; 3 Department of Mechanical and Materials Engineering, Faculty of Engineering, Universiti Kebangsaan Malaysia, Bangi, Selangor, Malaysia; Van Andel Institute, United States of America

## Abstract

Osteoporosis is becoming a major health problem that is associated with increased fracture risk. Previous studies have shown that osteoporosis could delay fracture healing. Although there are potential agents available to promote fracture healing of osteoporotic bone such as statins and tocotrienol, studies on direct delivery of these agents to the fracture site are limited. This study was designed to investigate the effects of two potential agents, lovastatin and tocotrienol using targeted drug delivery system on fracture healing of postmenopausal osteoporosis rats. The fracture healing was evaluated using micro CT and biomechanical parameters. Forty-eight Sprague-Dawley female rats were divided into 6 groups. The first group was sham-operated (SO), while the others were ovariectomized (OVx). After two months, the right tibiae of all rats were fractured at metaphysis region using pulsed ultrasound and were fixed with plates and screws. The SO and OVxC groups were given two single injections of lovastatin and tocotrienol carriers. The estrogen group (OVx+EST) was given daily oral gavages of Premarin (64.5 µg/kg). The Lovastatin treatment group (OVx+Lov) was given a single injection of 750 µg/kg lovastatin particles. The tocotrienol group (OVx+TT) was given a single injection of 60 mg/kg tocotrienol particles. The combination treatment group (OVx+Lov+TT) was given two single injections of 750 µg/kg lovastatin particles and 60 mg/kg tocotrienol particles. After 4 weeks of treatment, the fractured tibiae were dissected out for micro-CT and biomechanical assessments. The combined treatment group (OVx+Lov+TT) showed significantly higher callus volume and callus strength than the OVxC group (p<0.05). Both the OVx+Lov and OVx+TT groups showed significantly higher callus strength than the OVxC group (p<0.05), but not for callus volume. In conclusion, combined lovastatin and tocotrienol may promote better fracture healing of osteoporotic bone.

## Introduction

The aging population has persistently increased, resulting into more and more middle-aged and senile patients affected by age-related or postmenopausal osteoporosis [1].The prevalence of osteoporosis increases dramatically with aging in women, from 5% at 50 years old to 50% at 85 years old [2]. Thus, osteoporosis is becoming a major health problem as it is associated with compromised stability and reduced mobility of the patients [3].Osteoporosis is defined as a condition where the bone mineral density (BMD) falls below 2.5 standard deviations of peak mass as measured by Dual-Energy X-ray Absorptiometry (DXA) [4]. It can be characterized by low bone mass and microarchitectural deterioration of bone tissue, which may lead to bone fragility fracture [5].

Fracture healing is a complex process that occurs through a regulated cascade. It can be divided into three distinct phases; the reactive phase, reparative phase and remodeling phase. In the reactive phase, haematoma is formed between the fracture ends and rapidly seals off the fracture [6]. Then, the fracture haematoma will be replaced by granulation tissue and capillary ingrowths may occur rapidly. The fracture healing process is followed by the reparative phase, where formation of internal callus and deposition of lamellar bone will occur. Then, the callus will become mineralized to form a hard callus of woven bone. In the final or remodeling phase, the callus and vasculature will be remodeled to their normal shape and size [7]–[11].

Previous studies have shown that osteoporosis could lead to adverse consequences such as delayed fracture healing, disturbed callus formation with decreased bone mineral density (BMD), volume, microstructure, and biomechanical property [12]–[15]. Presently, no effective treatments are available for fracture healing [16].Thus, there is a need to find suitable agents and mode of delivery to speed up fracture healing of osteoporotic bone.

Osteoporosis drugs are commonly prescribed to prevent fractures, which is the principal aim of osteoporosis management [17]. Several anti-osteoporosis drugs such as strontium ranelate and parathyroid hormone (PTH) may support fracture healing as there are studies that have indicated their clinical benefits in promoting fracture healing in humans [16]. However, there are reports that anti-osteoporosis drugs such as bisphosphonates, denosumab and estrogen replacement therapy did not produce any significant effect on fracture healing [16], [18], and [19]. These agents were shown to suppress bone resorption activity in ovariectomized rats but they also caused secondary suppression of bone formation activity, resulting in poor bone remodeling [20]–[22]. Thus, the effects of anti-osteoporosis agents on fracture healing are controversial and more studies are needed to further elucidate their effects on bone healing. Studies should also be extended to other potential agents such as natural products and statins for fracture healing treatments.

Vitamin E is a lipid soluble vitamin which has shown potential in promoting fracture healing. It consisted of two types; tocopherol and tocotrienol. Generally, tocotrienol had higher antioxidant property that offered better bone protection compared to tocopherol [23], [24].Tocotrienol is a unique vitamin E which can be extracted from plants such as palm, rice bran and annatto bean. The annatto bean-derived tocotrienols, which contain 90% delta and 10% gamma tocotrienols, are the most active type of tocotrienols [25]. Previously, small daily intake of annatto tocorienol has been shown to reduce serum levels of cholesterol, triglyceride and LDL by 15 to 20% [26]. Annatto tocotrienols were also reported to have anti-cancer, anti-oxidant and anti-hyperglycemic properties [27]–[29]. In a study by Abdul-Majeed et al., [30], co-administration of annatto tocotrienols and lovastatin to ovariectomised rats have shown significantly higher bone formation and lower bone resorption. It was suggested that the combination of annatto tocotrienols and lovastatin may have synergistic or additive effects on bone metabolism. However, the tocotrienol doses may need to be adjusted to account for their low bioavailability, since they were not recognized by α-tocopherol transfer protein (a-TTP) for intake into the body [31].

Statin is a well-known anti-hyperlipidemic agent that inhibits 3-hydroxy-3-methylglutaryl coenzyme A (HMG CoA) reductase that catalyzes the conversion of HMGCoA to mevalonic acid in the cholesterol synthesis pathway. Statins have also pleiotropic actions in the cardiovascular, immune and nervous systems [32]. Lovastatin is a prodrug that is reversibly converted into active β-hydroxyacid form by carboxyesterase enzyme [33]. There are studies which reported that statins were able to increase bone formation and has potential for the treatment of osteoporosis and its complications by stimulation of bone morphogenetic proteins-2 (BMP-2) [34]. However, the oral dose of statins given to lower serum cholesterol may unlikely have any action on bones as they are metabolized in the liver, resulting into much lower concentration reaching the fracture site [35]. Therefore, high doses of oral statins are needed to reap the benefit of their anabolic actions on bone. However, high doses of statins were associated with liver failure, kidney disease and rhabdomyolysis [36].

In order to improve the bioavailability and effectiveness of tocotrienol and statin, a controlled drug delivery (CDD) system is required. Surgery to fix fractures provides access for drugs to be administered directly to the fracture site. CDD involved the use of formulation components to release a therapeutic agent at constant predictable rate *in vivo* when given through injected or non-injected route. The formulation is formed when a polymer (natural or synthetic), is combined with a drug or other active agent, where is then released from the material constantly over a long period [37]. In controlled drug delivery systems, the drug or active agent levels will remain constant between the desired maximum and minimum concentrations for an extended period of time [38]. The extended period may be anywhere from 24 hours (Procardia XL) to 1 month (Lupron depot) to 5 years (Norplant), depending on the formulation and the application used [37]. Since drugs are delivered locally at the target site, only small doses are required, which lower the risk of adverse drug reactions.

Structural characteristics and volumetric bone mineral density (BMD) for a callus can be measured using micro computed tomography (μCT). This quantitative method of analysis is non-destructive and in pre-clinical models of fracture healing, it can be followed by biomechanical testing to further determine the function of callus strength. By performing the micro CT evaluation and biomechanical testing, the changes in callus strength with treatment could be explained by changes in callus structure and BMD [39].

The present study was designed to determine whether controlled delivery of lovastatin and tocotrienol could promote fracture healing in postmenopausal osteoporosis rats by assessing the micro CT and biomechanical parameters. The findings from this study may provide an alternative agents and delivery systems for fracture healing of osteoporotic bone.

## Materials and Methods

### Animals and surgery procedure

Forty eight *Sprague Dawley* female rats, aged 3-months-old and weighing 200 to 250 g were used in this study. They were purchased from laboratory animal resources unit, Faculty of Medicine, Universiti Kebangsaan Malaysia (UKM). The rats were kept two per cage under 12-hour light-dark cycle and were given tap water *ad libitum*.

After one week of acclimatization, the rats were assigned randomly into 6 groups with 8 rats per group. The first group was sham-operated for surgical stress simulations while the other five groups were ovariectomised. Then, they were left untreated for two months to allow osteoporosis to develop in the ovariectomised rats. Fracture procedures were then performed on the right tibiae of all the rats using pulsed ultrasound and stabilized using plate fixation method [40]. Each rat was anaesthetized with intraperitoneal injection of Ketamine and Xylazil at 1∶1 ratio. Firstly, the operated-side of the leg was shaved with an electric shaver and sterilized with 70% alcohol, before incisions were made. An anterior-medial approach with an extension from the medial femur condyle to the middle of the tibia was used to gain access to the tibial bone. Then, without damaging the flexor and extensor muscles at the tibia, the proximal tibial third was prepared in an epiperiosteal manner. A 5-hole, 90° small T shaped titanium fixation plate XS (57-05140 Stryker Trauma, Selzach, Switzerland) was slightly pre-bent in the transversal part, and was fixed proximally with a 1.2 mm screw to the anterior-medial surface of the tibia.

**Figure 1:**
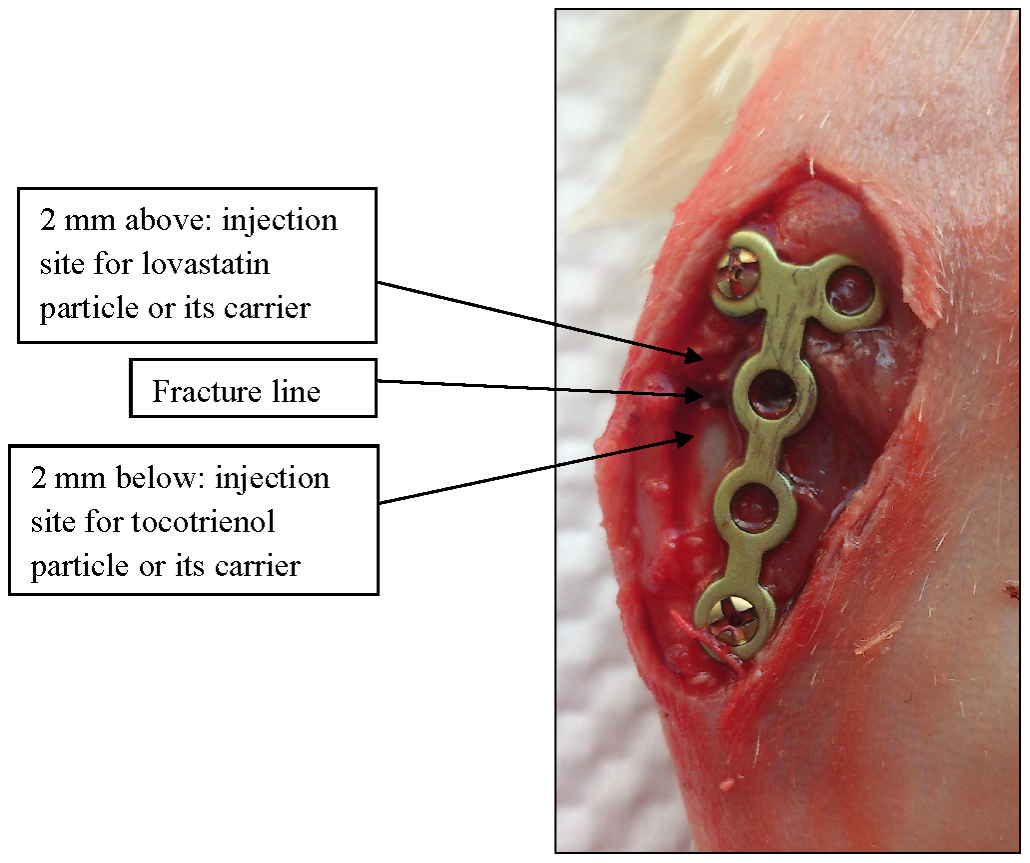
Specific sites of injection for lovastatin and tocotrienol particles. Injection sites were made at 2 mm above the fracture line and 2 mm below the fracture line for lovastatin and tocotrienol particles respectively.

During osteotomy, the plate was still in slightly pre-bent position. The osteotomy was performed at the metaphysis region using pulsed ultrasound (Mectron Medical Technology, Carasco, Italy) by setting the tool for spongious bone power outputs. This tool produces specific ultrasound frequency modulation ranging from 22,000 to 35,000 Hertz and thus required very limited manual pressure to guide the osteotomy precisely. This tool would only cut hard materials, therefore, any damages to muscles, tendons, and nerves would be avoided. Confirmation of a complete fracture with osteotomy was made through naked eye observation. After the osteotomy, the slightly pre-bent plate was placed in its correct position and fixed with another 1.2 mm screw distal to the anterior-medial surface of the tibia to stabilize the fracture. Immediately, two single injections of treatments (0.05 ml per 100 g body weight) were delivered into the muscles near the fracture site. Specific site of injections have been assigned. Lovastatin particle or its carrier was injected 2 mm above the fracture line, while tocotrienol particle or its carrier was injected 2 mm below the fracture line (Fig. 1). The skin incision was closed with non-absorbable suture. Each rat received injections of Baytril 5% (0.1 mg/kg body weight every 24 hours for 5 days) as antibiotic and buprenorphine (0.1 mg/kg of bodyweight every 12 hours) as analgesic. Iodine solution was applied to the site for post-operative antiseptic. Daily oral gavages of treatment were started one day after the fracture for 4 weeks. This study has been approved by Universiti Kebangsaan Malaysia Animal Ethical Committee (FP/FAR/2012/NAZRUN/21-NOV/472-NOV2012-MAR2014).

### Categorization of rats and treatments

The sham-operated (SO) and ovariectomised control (OVxC) groups were given two single injections of lovastatin carrier and tocotrienol carrier. The estrogen group (OVx+EST) was given daily oral gavages of Premarin (64.5 µg/kg) and two single injections of lovastatin carrier and tocotrienol carriers. The Lovastatin treatment group (OVx+Lov) was given two single injections of 750 µg/kg lovastatin particles and tocotrienol carrier. The tocotrienol group (OVx+TT) was given two single injections of 60 mg/kg annatto tocotrienol particles and lovastatin carrier. The combined treatment group (OVx+Lov+TT) was given two single injections of 750 µg/kg lovastatin particles and 60 mg/kg annatto tocotrienol particles. All the groups were given daily oral gavages of deionised water, except for the group receiving oral Premarin. Following four weeks of treatment period, the rats were euthanized and the fractured tibiae were extracted. The plates and screws were then removed. The tibiae were wrapped in gauze that have been immersed in phosphate buffered saline (PBS), and stored at −70°C until analyzed.

### Preparation of particles

Lovastatin particles were prepared according to Garrett et al., [41]. Briefly, 2.5 ml of 100 mg/ml Poly-(DL-lactide), viscosity of 0.26 to 0.54 (DURECT Corporation, Birmingham, Alabama, USA) was mixed with agitation with 1 ml of 50 mg/ml lovastatin in acetone. Then, 2.5 ml of water 1% polyvinyl alcohol was added in a dropwise fashion with continuous stirring. The mixture was stirred at room temperature for 5 hours to remove the remaining solvent and unloaded drugs. Next, the substance was centrifuged at 10,000/rpm and repeatedly washed with saline. The sediment (resultant lovastatin particle) was collected and dried under reduced pressure overnight. The particles were resuspended in saline for immediate use and freeze-dried for long term storage.

Tocotrienol particles were prepared by solvent-evaporation method according to Wakiyama et al. 1981 [42] and Urata et al. 1999 [43]. Briefly, about 10 mg of delta-tocotrienol and 50 mg of poly-(DL-lactide-co-glycolide) were dissolved in 2 ml of dichloromethane. Then, the solution was cooled to 4°C for about 1 hour to form crosslink, before being dispersed in 0.1 polyvinyl alcohol solutions using magnetic stirrer. The stirring was continued for 2 hours at room temperature to evaporate off the methylene chloride. The mixture was filtered using 0.45 µm filter, washed with distilled water, and dried under reduced pressure for 2 days. Finally, the resultant tocotrienol particles were collected and suspended in a solution of 0.5% carboxymethyl cellulose and 0.1% Tween 80 before injected to the rats. The carriers of lovastatin and tocotrienol were prepared using the same preparation methods, but without the addition of lovastatin and tocotrienol respectively.

### Micro-computed tomography (Micro-CT)

After thawing, each fractured tibia was re-wrapped with PBS-moisted gauze and was fitted vertically in the μCT specimen tube. In order to acquire images, μCT 80 scanner (SCANCO Medical, Switzerland) required measurement protocols such as energy settings of 70 kV and 114 mA. High definition resolution of 5 µm voxel size was used to obtain better image. Following reconstruction, the fracture line was identified by viewing multiple orthogonal slices. The region of interest was determined as 150 axial slices above and below the fracture line. The μCT based measurements for the region of interest (ROI) of each callus were determined manually using SCANCO image analysis software.

The parameters included were total callus volume (TV_callus_), mineralized callus volume (BV _callus_), ratio of the mineralized tissue within the total callus volume (BV_callus_/TV _callus_) and the volumetric BMD of the mineralized tissue comprising the callus (mBMD_callus_). In order to obtain such measurements, a contour was made surrounding the perimeter of each callus slice. The same settings of auto-contouring feature with the Scanco software were used for all the callus slices. In the presence of a gap in the callus, manual correction was done at the contour.

**Figure 2:**
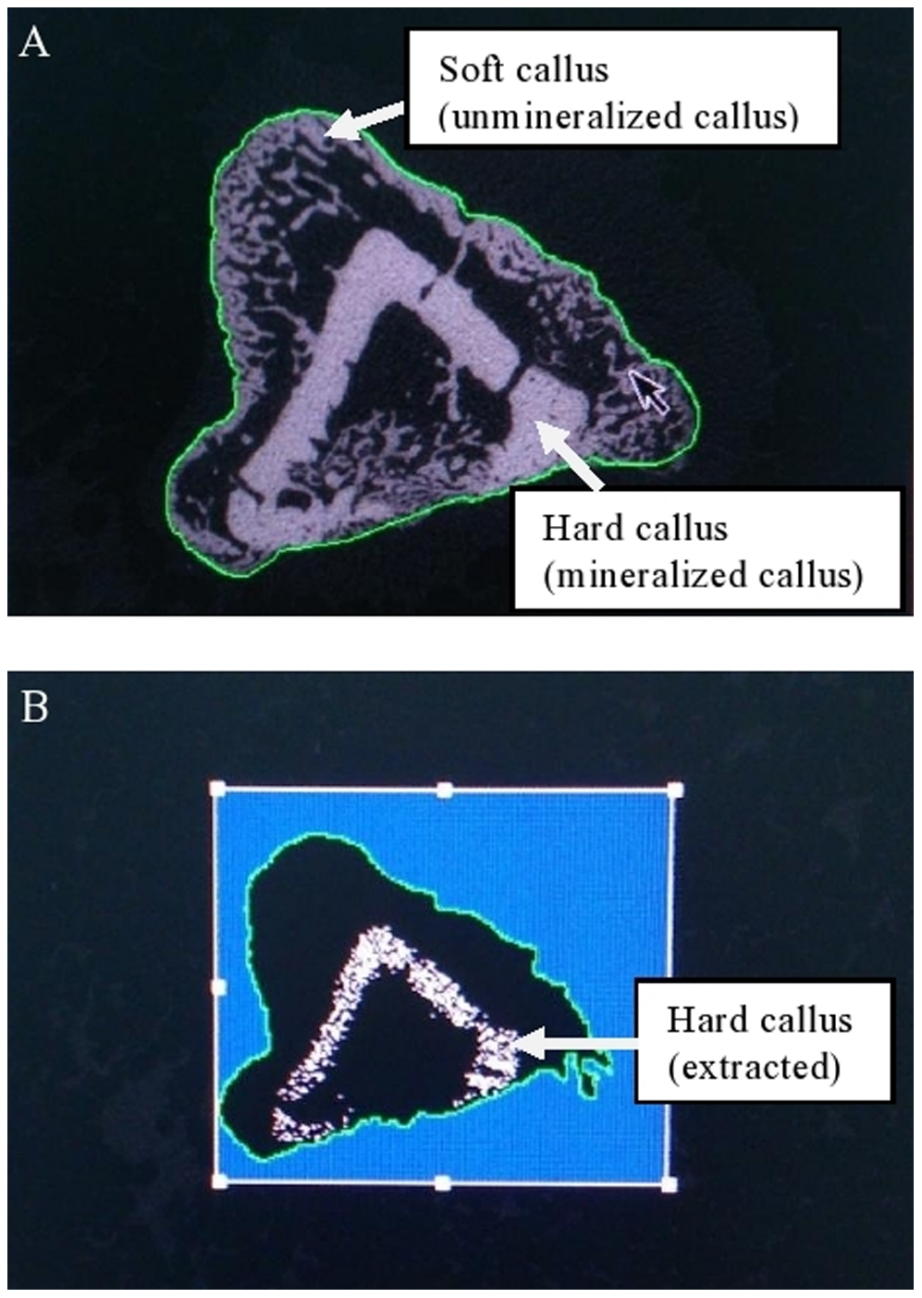
Extraction of mineralized callus from total callus area. a) Contour line was made to set for total callus area; b) Extraction of mineralized callus by applying threshold of 421 mgHA/cm^3^. Higher mineralized callus volume extracted from the total callus area showed better improvement in fracture healing.

The contour line of each callus represented the entire region inside the contours (TV _callus_).The mineralized callus (BV_callus_) was extracted by using global thresholding of 421 mgHA/cm^3^ (Fig. 2), Guassian noise filter of 2 and support of 2. The same global thresholding settings were used for all analyses.

### Biomechanical testing

The tibiae which have been stored in phosphate-buffered saline at −70°C were thawed at room temperature before the biomechanical test. The tibia was subjected to a three-point bending test on Instron Universal Testing Machine (Instron Microtester 5848, Instron Corp USA) with Bluehill software. They were kept moist at all times during the preparation procedure.

**Figure 3:**
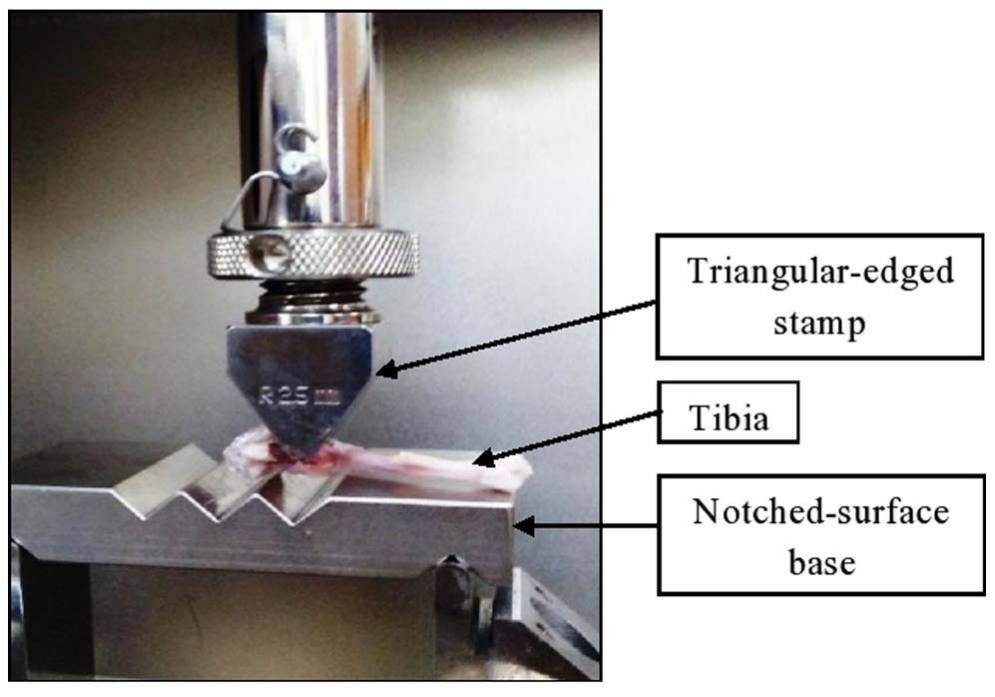
Configuration of three-point bending test. The configuration promoted a stable three-point contact where the tibia would be able to lengthen along diaphyseal axis.

Three-point bending configuration was developed according to Stuermer *et al.* 2006 [44]. The configuration consisted of a blunt triangular edge on the top and an aluminum block with notches as a base (Fig. 3). Due to its S shape, the tibia was rested on the aluminum base with the two condyles at the notches and the distal diaphysis on the other side. This would result in a stable three-point contact, as the tibia could not slip, but would be able to lengthen along the diaphyseal axis. The size of the calluses was checked to make sure that they were not large enough to compromise their positioning. A careful observation was made to detect the fracture line, as the top triangular edge would be directed at the former osteotomy position before the biomechanical test could be initiated. During the test, load was driven down onto the metaphyseal tibia at the point of the former osteotomy at the speed of 5 mm/min until it refractured. The load, stress, and strain parameters were recorded by the software. These parameters are important as the loads and forces required to deform the fractured bone could be measured. Graph of stress against strain was plotted, where Young's Modulus could be derived from the curve gradient. The Young's Modulus measurement is also an imperative parameter because it represents the stiffness of the callus.

### Statistical analysis

Results were analyzed using Statistical Package for Social Science software (SPSS version 20.0) and were expressed as mean ± standard error mean (SEM). The data was tested for normality using Kolmogorov-Smirnov test. For normally distributed data, the statistical test used were the analysis of variance (ANOVA), followed by post-hoc analysis of tukey's HSD test. The level of significance was taken as p<0.05.

## Results

### Micro-CT

**Figure 4:**
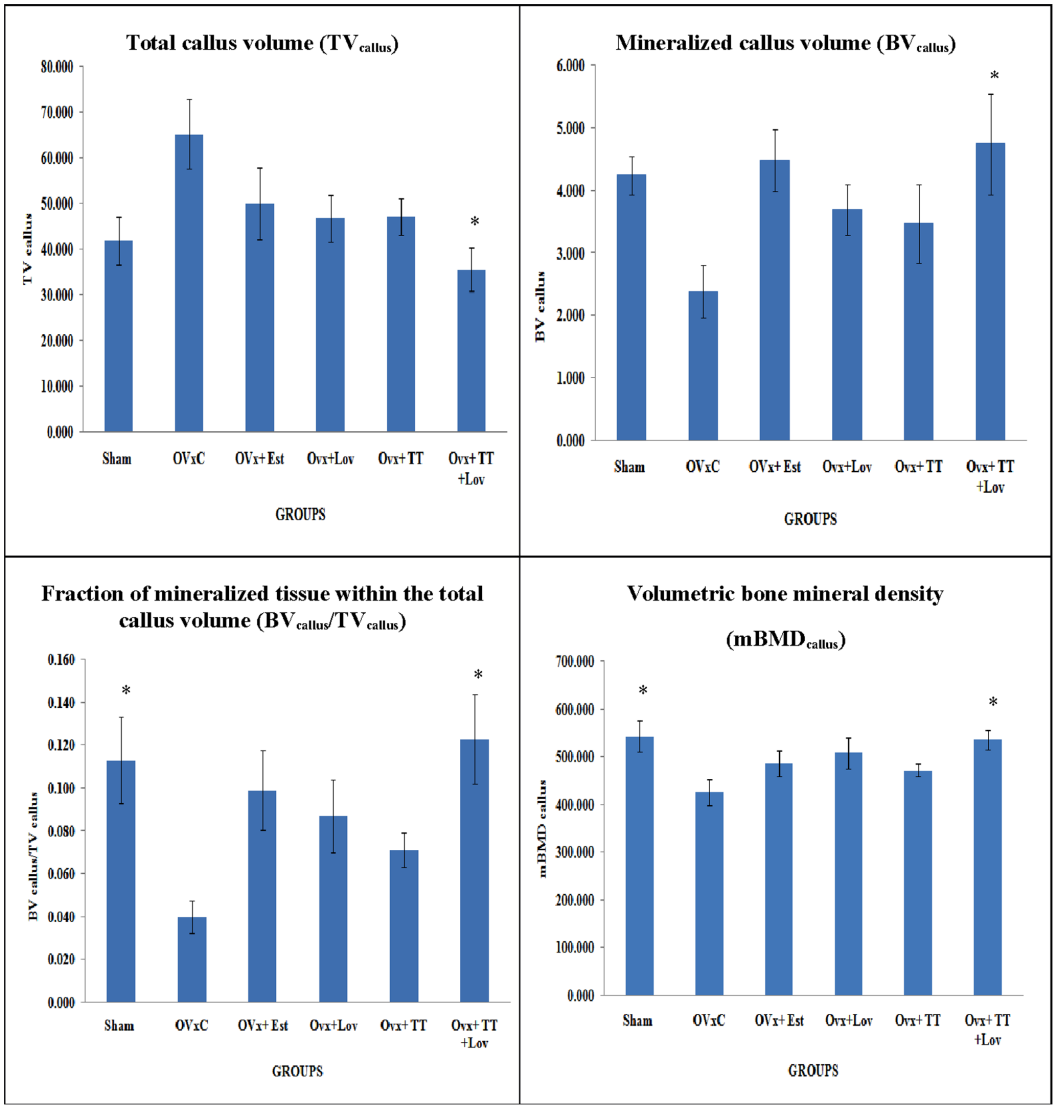
3-D structure of mineralized callus after undergoing global thresholding at 421 mgHA/cm^3^ for micro-CT reconstruction. Sham:sham-operated group, OVxC:ovariectomized control group, OVx+Est:ovariectomized+ Estrogen, OVx+Lov:ovariectomized+Lovastatin, OVx+TT:ovariectomized+Tocotrienol, OVx+TT+Lov:ovariectomized+Tocotrienol+Lovastatin.

**Figure 5:**
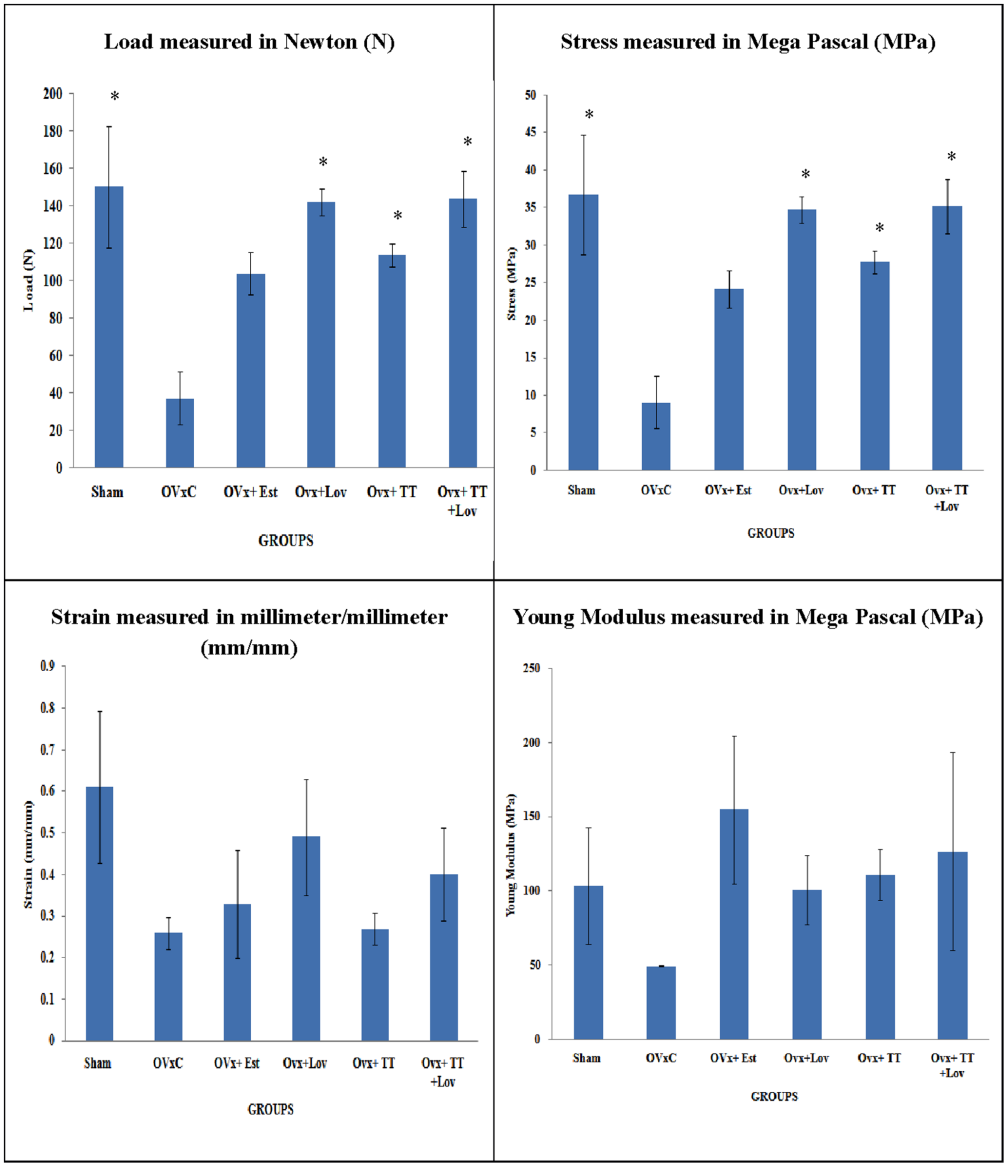
Total callus volume, Mineralized callus volume, Fraction of mineralized tissue within the total callus volume, Volumetric bone mineral density for all the groups after 4 weeks of treatment. Sham: sham-operated group, OVxC:ovariectomized control group, OVx+Est:ovariectomized+ Estrogen, OVx+Lov:ovariectomized+Lovastatin, OVx+TT:ovariectomized+Tocotrienol, OVx+TT+Lov:ovariectomized+Tocotrienol+Lovastatin. *Indicates a significant difference (p<0.05) compared with the ovariectomized control group (OvxC). The data were expressed as mean ±SEM.

Micro-CT images of the 3-D structure of mineralized callus that were formed after micro-CT reconstruction is shown in (Fig. 4). Based on the images, the ovariectomized control (OVxC) group had the least amount of mineralized callus compared to the other groups. The combination (Ovx+TT+Lov) group showed a more complete formation of mineralized callous compared to the other groups (Fig. 4). The Ovx+TT+Lov had significantly lower TV_callus_ compared to the OvxC group (p<0.05). Apart from that, the Ovx+TT+Lov group had significantly higher BV_callus_ and BV_callus_/TV_callus_ compared to the OvxC group (p<0.05). Besides that, the sham-operated (Sham) group also showed significantly higher BV_callus_/TV_callus_ compared to the OvxCgroup (p<0.05). For mBMD measurement, the Ovx+TT+Lov and Sham groups showed significantly higher mBMD compared to the OvxC group(p<0.05).There were no other significant differences among the various treatment groups for all the micro-CT variables (Fig. 5).

### Biomechanical testing

**Figure 6:**
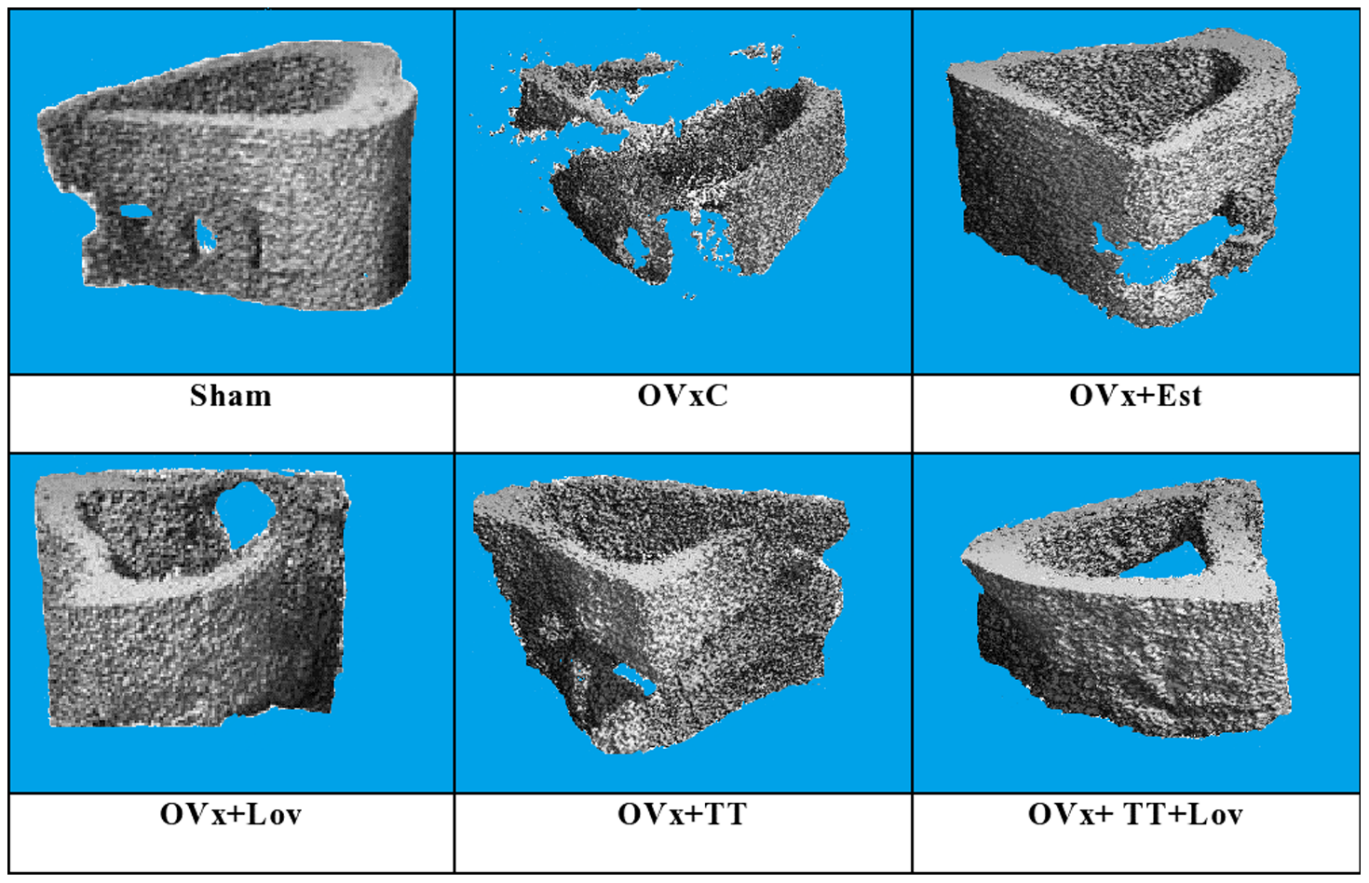
Load, Stress, Strain and Young Modulus for all the groups after 4 weeks of treatment. Sham: sham-operated group, OVxC:variectomized control group, OVx+Est:ovariectomized+ Estrogen, OVx+Lov:ovariectomized+Lovastatin, OVx+TT:ovariectomized+Tocotrienol, OVx+TT+Lov:ovariectomized+Tocotrienol+Lovastatin. *Indicates a significant difference (p<0.05) compared with the ovariectomized control group (OvxC). The data were expressed as mean ±SEM.

The strength of the callus was assessed using biomechanical testing. The calluses of the Sham, OVX+Lov, OVX+TT, and OVX+TT+Lov groups were able to handle significantly greater load compared to the OVXC group (p<0.05). The load parameter for the calluses of the OVX+Est group were not significantly different compared to the OVXC group. Apart from that, the calluses of the Sham, OVX+Lov, OVX+TT, and OVX+TT+Lov groups were able to handle significantly greater stress compared to the OVXC group (p<0.05). The stress parameter for the calluses of the OVX+Est group were not significantly different compared to the OVXC group. There were no significant differences for strain and Young modulus parameters between all the groups (Fig. 6). Besides that, no other significant differences were detected among the various treatment groups.

## Discussion

Fracture callus is a complex structure made up of fibrous tissue, cartilage, and immature fibre bone that are formed during the intermediate phase of fracture healing [45]. Evaluation of fracture callus was performed at one-time point (4 weeks of post fracture), during which the healing processes was in the intermediate phase, allowing effects of fracture healing to be observed. Evaluation of fracture healing during early or late phase may result into unpronounced healing or complete healing respectively.

One of the basic micro CT parameters is TV_callus_, which is defined as the sum of volumes of all mineralized tissue and non-mineralized tissue within the region of interest [46].The ovariectomized control (OVxC) group showed the highest TV_callus_, which may be contributed by the high content of non-mineralized tissue. This was confirmed by the persistently low mineralization parameters for the OVxC group, including BV_callus_, BV_callus_/TV_callus_ and mBMD_callus_. These findings were consistent with a previous study by Shuid *et al*.,(2010) which reported that the total callus volume in ovariectomized-control rats was higher than sham-operated rats at 8 weeks post-fracture [47].The amount of cartilage in the calluses of the OVxC group was much greater than other groups, exhibiting characteristic of immature calluses. This corresponded to the middle phase of fracture healing, where the total callus volume was high and mainly composed of immature soft callus. In a previous study by Mehta *et al.* (2013) [48], it was reported that high total callus volume was associated with lower strength and stiffness, and do not correspond to better healing. Thus, in the present study, the OVxC group may have exhibited poor fracture healing due to estrogen deficiency, which delayed mineralization process or endochondoral ossification of soft callus in the fractured tibia. Estrogen deficiency may also cause an overproduction of reactive oxygen species, which promoted osteoclast activity [49] and delaying the fracture healing rate. Although not statistically different when compared to the OVxC group, TV_callus_ were still lower in the Sham, OVx+Est, OVx+Lov and OVx+TT groups, which may indicate some improvement in osteoporotic fracture healing with either of the treatment. Only the combined treatment group (OVx+TT+Lov) showed significantly lower TV_callus_ compared to the OVxC group. The lower TV_callus_ in the OVx+TT+Lov group indicated that most of the soft callus had been replaced by mineralized callus and thus were mainly composed of hard callus of woven bone. This was consistent with the mineralized callus volume (BV_callus_) parameter, where the OVx+TT+Lov group showed significantly higher BV_callus_ than the OVxC group. The callus formed in the OVx+TT+Lov group showed faster healing, probably due to the synergistic or additive effect of lovastatin and tocotrienol particles. They may have suppressed the activity of HMG-CoA reductase in the mevalonate pathway via two different mechanisms, where statins inhibited the enzyme activity through competitive inhibition, while tocotrienols modulated the intracellular mechanism of controlled degradation of HMG-CoA reductase protein [50], [51]. Inhibition of the HMG-CoA reductase protein activity reduced the availability of mevalonate, and thus reductions of prenylated proteins such as RhoA. The inhibition of RhoA promoted bone morphogenetic proteins (BMP)-2-induced osteoblastic differentiation processes and accelerate bone formation [52].These events may improve fracture healing as indicated by the significantly higher mineralized callus.

The OVx+TT+Lov and Sham groups showed significantly higher BV_callus_/TV_callus_ when compared to the OVxC group. The Sham group, that represented the fracture healing of normal bone showed a good progression of fracture healing. The intact ovaries in the sham-operated rats continued to secrete estrogen, thus improving the quality of callus formed during fracture healing. Estrogen has been shown to promote mineralization of soft callus that led to the formation of a mature callus [53]. As expected, the Sham group showed a significantly higher BV_callus_/TV_callus_ compared to the OVXc group. The OVx+TT+Lov group showed the highest BV_callus_/TV_callus_, which indicated high content of mineralized tissues within the callus. Therefore, combination of lovastatin and tocotrienol provided the most effective treatment for fracture healing of osteoporotic bone. The other groups i.e. OVx+Est, OVx+Lov and OVx+TT groups also had higher BV_callus_/TV_callus_ than the OVxC group but were statistically insignificant.

According to Nyman *et al.* (2009), the mBMD_callus_ has the strongest correlation with callus strength, in which it is directly related to strength and stiffness [39]. Osteoporosis impaired fracture healing, resulting in poorly mineralized callus, as shown by the low mBMD_callus_ in the OVxC group_._ Therefore, the callus formed was weak compared to the fracture healing of normal bone as in the Sham group. The fractures in the Sham and OVx+TT+Lov groups healed with significantly higher mBMD_callus_ compared to the OVxC group. This showed that combined targeted delivery of lovastatin and tocotrienol to the fracture site were able to promote fracture healing of osteoporotic bone by restoring the callus mineralization until they were at par with fracture healing of the Sham group. However, single treatment with tocotrienol or lovastatin and even the standard treatment with estrogen were not able to cause significant improvement in mBMD_callus_ during fracture healing.

Regardless of the type of callus formed during fracture healing, what matters most is the strength it offered to prevent fracture recurrence. In the biomechanical evaluation, as expected, the calluses of the OVx+TT+Lov group were able to handle significantly higher load and stress compared to the OVxC group. However, the calluses of both the single treatment groups (OVx+Lov and OVx+TT) were also able to handle significantly higher stress and load compared to OVxC. The callus strength of estrogen-treated rats (OVx+Est group) remained insignificant compared to ovariectomised-control rats (OVxC group). This indicated that individually, lovastatin and tocotrienol may improve the mechanical strength of callus although it was not accompanied by significant improvements in the callus microstructure during fracture healing. This finding was supported by a previous study by Sharlina *et al*. (2012), which reported that oral supplementations of palm tocotrienol could improve the callus strength during fracture healing of osteoporotic bones even though there was no radiological improvement seen [54].

Similarly, some of the micro-CT changes of the calluses were not accompanied by significant changes in their biomechanical strength. These may be explained by the fact that bone strength is not only determined by its structure or volume. It also depends on the morphology and intrinsic material properties that determine the orientation and arrangement of newly-formed bone [55].

Furthermore, micro-CT is a very sensitive tool that determines both the structural characteristics and volumetric bone mineral density [39]. Since fracture heals in a progressive manner, it may affect these parameters at different trend or pattern based on the aspects that were measured. To date, there is no study on local delivery of tocotrienol to the fracture site using targeted drug delivery system. As for lovastatin particle, Wang *et al*. (2010) has also reported that injection of controlled delivery of lovastatin adjacent to the fracture site to NF1-knockout mice had increased the callus strength by 23% compared to calluses of wild type mice [56].

In this present study, oral supplementations of estrogen for 4 weeks failed to improve any micro CT or biomechanical parameters, and thus did not seem to promote the fracture healing of ovariectomized rats. Contradictorily, in a study by Estai *et al.*(2011) [57], osteoporotic femoral-fractured rats receiving estrogen for 6 weeks showed greater remodeling of the bone calluses compared to ovariectomized control rats. However, there were several differences between the present study and that of Estai *et al.* Firstly; the dose of estrogen used was higher, at 100 µg/kg compared to 64.5 µg/kg in the present study. The fracture methods and type of fracture were also different. Estai *et al*. had induced mid-diaphyseal fracture of the femur, as compared to the metaphyseal fracture of the tibia in the present study. Osteoporotic fractures occur more commonly in areas of trabecular bone such as metaphysis. This is because trabecular bone has higher bone turnover than cortical bone. Thus, metaphyseal fracture in the present study more closely resembles osteoporotic fracture.

Cao *et al.* 2002 [58] reported that estrogen treatment for 6 and 16 weeks to osteoporotic rats with femoral fracture had suppressed bone turnover and did not improve fracture healing. In postmenopausal women with osteoporosis, estrogen has been shown to prevent loss of bone mass [59], by suppressing bone resorption activity. However, it may also cause secondary suppression of bone formation activity, which may adversely affect bone remodeling [18]–[21]. This could be the reasons why estrogen in the present study, failed to improve fracture healing.

In conclusion, lovastatin and tocotrienol particles administered directly to the fracture site using controlled drug delivery system, could improve the mineralization and strength of callus formed during fracture healing of osteoporotic bone. Single treatment of either lovastatin or tocotrienol using the same delivery system, did not appear to affect callous mineralization, but could improve their strength. Further studies are required to determine the mechanisms of fracture healing with controlled delivery of lovastatin and tocotrienol.
